# Supplementary material for: Enhanced Systemic Humoral Immune Response Induced in Mice by Generalized Modules for Membrane Antigens (GMMA) Is Associated with Affinity Maturation and Isotype Switching
Source: Vaccines (Basel). 2023 Jul 8;11(7):1219. doi: 10.3390/vaccines11071219 (PMC10384117; doi:10.3390/vaccines11071219)
Supplement: Supplementary file 1 [file vaccines-11-01219-s001.zip › vaccines-2454525-supplementary.pdf]

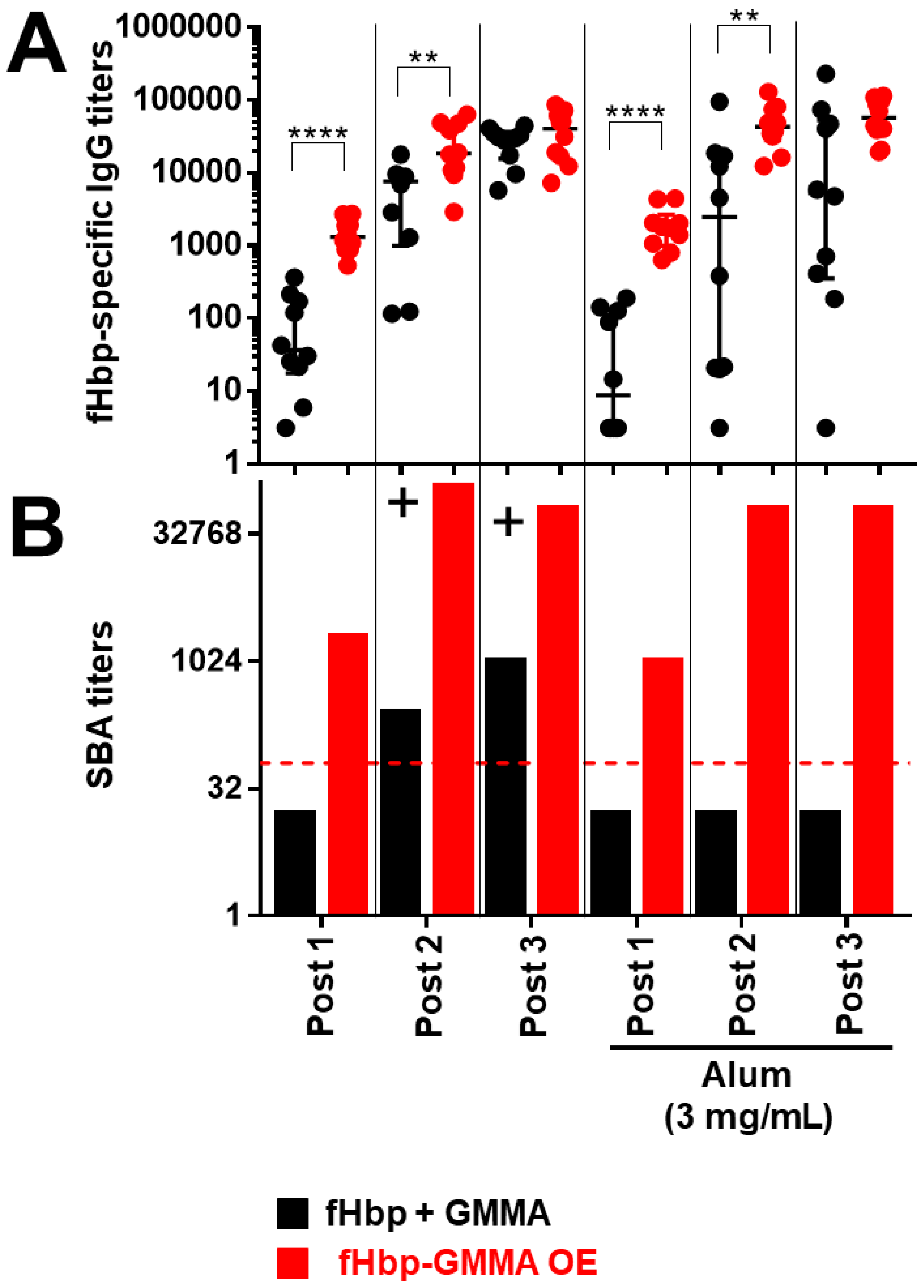

Figure S1. GMMA carrier effect along the different time points of the immunization schedule. CD1 mice were immunized intramuscularly three times with fHbp physically mixed to MenB GMMA (fHbp + GMMA; black dots or columns) or with MenB GMMA overexpressing fHbp (fHbp-GMMA OE; red dots or columns), either adsorbed or not on Alum, at the same doses of fHbp and GMMA. MenB GMMA used in physical mixture were deleted by fHbp. The dosages of fHbp and GMMA in physical mixture (as protein content) were 0.1 µg and 1.9 µg, respectively, whereas the dose of GMMA overexpressing fHbp (as protein content) was 2 µg (containing 0.1 µg of fHbp). Alum was used at 3 mg/ml, as indicated. Sera were collected the day before the first, the second (Post 1), and the third (Post 2) immunization and two weeks after the third (Post 3) immunization. Mouse

sera were analyzed by ELISA and SBA assays. A. fHbp-specific total IgG titers measured in individual mouse sera (dots) Post 1, Post 2, and Post 3. Data are reported using base-10 logarithmic scale (y-axis). B. SBA titers against UK320 MenB strain (reference for fHbp in the killing assay) measured in Post 1, Post 2, and Post 3 pooled mouse sera from each group of the study. Data are reported using base-2 logarithmic scale (y-axis). The red dotted line set at the SBA titer of 64 represents the threshold of significance for the assay to unequivocally establish a killing activity. An increase of at least 8-fold (+) in SBA titer between two serum pools is considered biologically significant. IgG ELISA titers were analyzed by GraphPad applying the two-tailed non-parametric Mann–Whitney test. The titers are graphically represented with median and interquartile ranges. **** *p* value: <0.0001. ** *p* value: < 0.01.

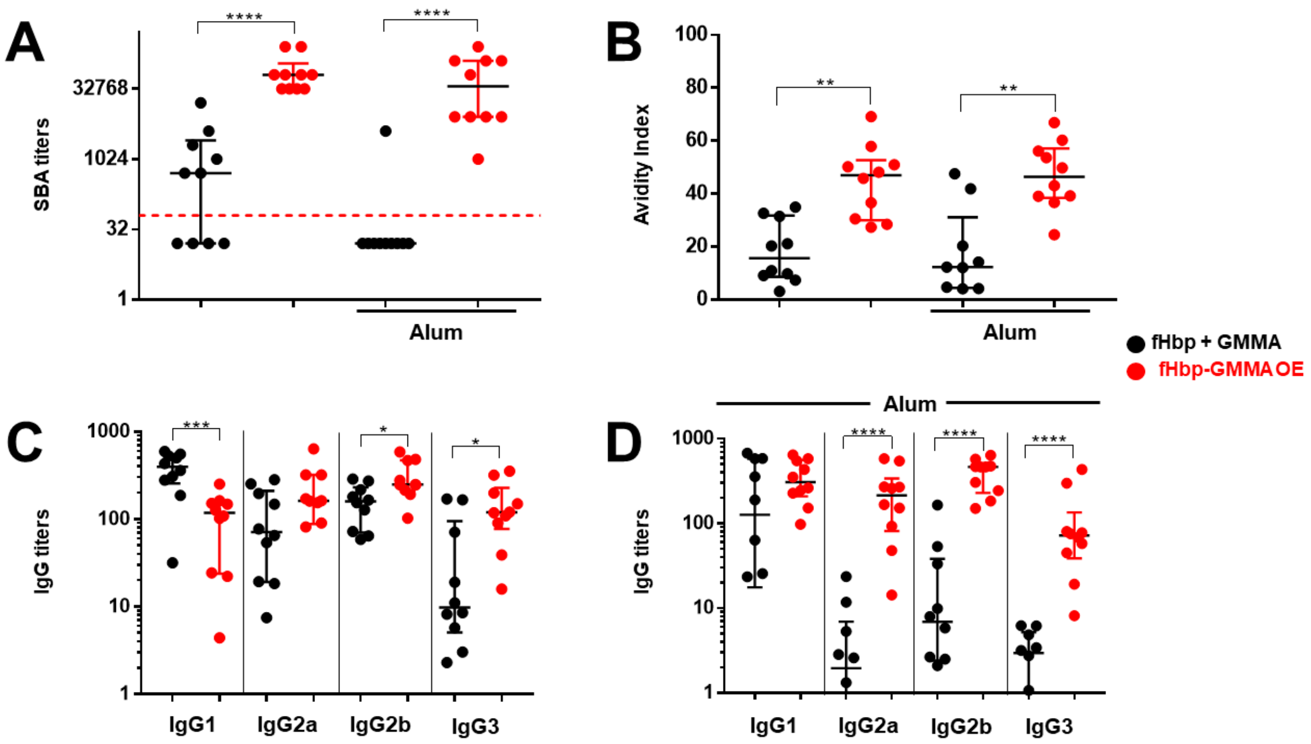

Figure S2. The superior functionality of the humoral immune response induced immunizing with GMMA bearing an antigen on the vesicle surface was associated with affinity maturation and isotype switching. Same experiment described in Figure S1. Analysis of Post 3 sera of mice immunized with fHbp + GMMA physical mixture (black dots) or GMMA overexpressing fHbp (red dots), with and without Alum, as indicated. A. SBA titers against UK320 MenB strain (reference for fHbp in the killing assay) measured in individual mouse sera. Data are reported as base-2 logarithmic scale (y-axis). B Avidity Index of fHbp-specific IgG, measured in individual mouse sera. Data are reported as linear scale (y-axis). C, D. Titers of fHbp-specific IgG subclasses (IgG1, IgG2a, IgG2b, and IgG3) measured in individual mouse sera immunized in (C) absence or (D) presence of Alum. Data are reported as base-10 logarithmic scale (y-axis). The red dotted line set at the SBA titer of 64 represents the threshold of significance for the assay to unequivocally establish a killing activity. IgG ELISA titers were analyzed by GraphPad, applying the two-tailed non-parametric Mann–Whitney test. The titers are graphically represented with median and interquartile ranges. **** *p* value: <0.0001. *** *p* value: <0.001. ** *p* value: < 0.01. * *p* value: < 0.05.
